# Advances in Colorectal Cancer Cell Biology and Clonal Evolution

**DOI:** 10.3390/ijms27020953

**Published:** 2026-01-18

**Authors:** Sopozme Toghey, Elizabeth J. Harvey-Jones, Jonathan D. Towler, Charlotte J. H. Hafkamp, Irene Y. Chong

**Affiliations:** 1 Department of Radiotherapy, Royal Marsden Hospital NHS Foundation Trust, Fulham Road, London SW3 6JJ, UK; s.toghey@nhs.net (S.T.); elizabeth.harveyjones@rmh.nhs.uk (E.J.H.-J.);; 2 National Institute for Health and Care Research (NIHR) Biomedical Research Centre, The Royal Marsden NHS Foundation Trust and The Institute of Cancer Research, 123 Old Brompton Road, London SW7 3RP, UK; 3 The Breast Cancer Now Toby Robins Research Centre, The Institute of Cancer Research, London SW3 6JB, UK; 4 Department of Radiation Oncology, Amsterdam University Medical Center, De Boelelaan 1118, 1081 HV Amsterdam, The Netherlands; 5 Honorary Faculty, The Institute of Cancer Research, London SW3 6JB, UK

**Keywords:** colorectal, transcriptional heterogeneity epigenetic regulation, cell biology clonal evolution

## Abstract

Colorectal cancer (CRC) develops through evolutionary processes involving genomic alterations, epigenetic regulation, and microenvironmental interactions. While traditionally explained by the stepwise accumulation of driver mutations, contemporary evidence supports a ‘Big Bang’ model in which many early-arising clones expand simultaneously to establish extensive heterogeneity. We reviewed recent studies employing spatially resolved multi-omic sequencing of tumour glands combined with computational modelling. These approaches enable high-resolution reconstruction of clonal architecture, transcriptional states, and chromatin accessibility. Findings show that although early clonal mutations shape tumour expansion, gene expression variability can be independent of genetic ancestry and instead reflects phenotypic plasticity driven by microenvironmental cues. Epigenomic analyses identified recurrent somatic chromatin accessibility alterations in promotors and enhancers of oncogenic pathways, frequently in the absence of DNA mutations, suggesting alternative mechanisms of gene regulation. Immune-focused studies demonstrated that early silencing of antigen-presenting genes and loss of neoantigens facilitate immune escape despite active surveillance. CRC is shaped by an interplay of genome, epigenome, and immune evolution, with non-genetic mechanisms and tumour plasticity emerging as important drivers of progression and therapeutic resistance.

## 1. Introduction

According to the International Agency for Research on Cancer (IARC) GLOBOCAN 2020 estimates, there were around 1.93 million new cases of colorectal cancer in 2020 [1]. In the last decade, the incidence of colorectal cancer has remained stable for patients over the age of 50 in high-income countries whilst increasing in middle to low-income countries [2]. However, young onset CRC (typically defined as diagnosis before the age of 50) has increased globally [3,4] emphasising the importance of understanding the cellular processes that underpin this disease.

**Figure 1:**
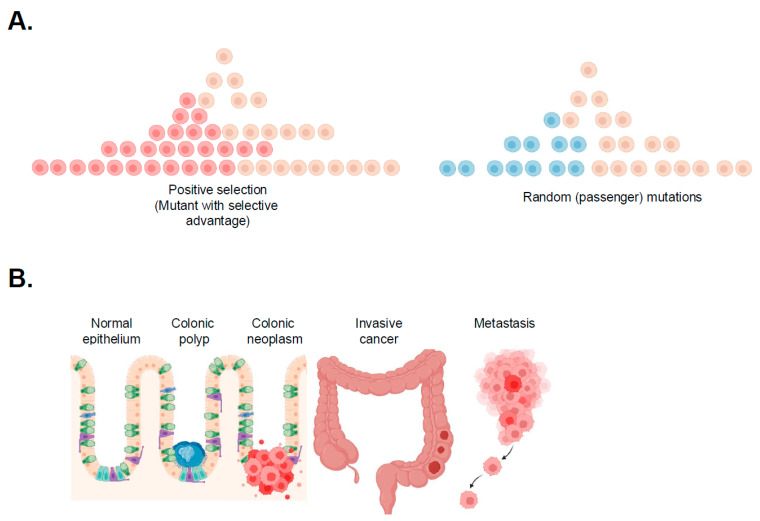
Neoplastic transformation of CRC and progression. (**A**) Schematic clonal expansion—Dynamics of somatic evolution in a heterogenous cell population. Positive selection is non-random, generating signatures of ‘clonal outgrowth’ (**left** panel). Random (passenger) mutations are largely stochastic (**right** panel) (adapted from [8]). (**B**) Classical steps of early neoplastic transformation in colorectal cancer (adapted from [12]).

CRC development is an evolutionary process where the cancer genome is shaped by positive selection, random mutation, and drift [5,6]. In the case of positive selection, the expansion of a cancer clone is caused by acquiring a ‘driver’ mutation that leads to an increased growth advantage [7]. In contrast, random mutations, or ‘passengers’, have no effect on a cell’s phenotype and are more abundant than ‘drivers’. On the other hand, drift occurs at random and arises when one clone has more offspring than another [8] [Figure 1A]. In practice, the distinction between neutral passenger mutations and selectively advantageous sub-clonal drivers is not based on a fixed threshold but inferred statistically [8,9]. Computational approaches assess whether the frequency of a mutation deviates from patterns expected under neutral tumour growth and consider its presence across tumour sites and whether it affects known cancer driver genes. A private mutation may represent either a neutral passenger or an early driver that has not yet expanded, and these scenarios are usually distinguished using a multifaceted approach rather than a single cut-off [10,11].

Initial cancer transformation events have long been thought to result from the stepwise accumulation of driver alterations [5,12,13,14] (Figure 1B). Through whole-genome sequencing, the mutational landscape of over 2000 colorectal cancers have recently been described [15]. Bulk sequencing of tumours has led to the valuable definition of new molecular subgroups [16], a spectrum of mutational profiles, including the Escherichia coli colibactin in rectal cancers and the SBS signature, which suggests that diet or smoking is a risk factor [15]. However, growth dynamics of established tumours has historically been poorly understood, and recent studies have shown that DNA mutations alone do not fully explain malignant transformation, suggesting a significant role for non-genetic influences such as epigenetic regulation and tumour microenvironment interactions [17,18,19].

Advancements in next-generation sequencing assays, computational analyses, as well as novel mathematical modelling have unravelled new insights into colorectal cancer cell biology, including the ability of cancer cells to change their behaviour in response to microenvironmental cues [20,21,22,23,24,25]. In contrast to bulk sequencing, the profiling of individual colorectal tumour glands allows the dissection of sub-clonal architecture at single-clone resolution, since neighbouring cells within a gland share a common ancestry [26,27,28,29,30]. Spatial profiling, that is, multi-omic sequencing of glands at different parts of the tumour, allows the in-depth study of intratumuor heterogeneity patterns and clonal evolution within an individual CRC [28]. In addition, focused study on how the epigenome interacts with the genome allows us to understand how physical access to DNA plays an essential role in establishing and maintaining cellular identity [19,31]. In this review, we consider concepts related to tumour plasticity, the co-evolution of the genome and epigenome, as well as how tumours interact with the immune microenvironment within the context of CRC.

## 2. Colorectal Cancer Pathogenesis and Molecular Classification

### 2.1. CRC Pathogenesis and Progression

Our knowledge of the development and evolution of CRC has grown with the application of increasingly sophisticated sequencing techniques and the ability to track CRC progression with multiple biopsies and at different time points of tumour development [32]. Approximately 70% of CRCs originate from adenomatous polyps that have become hyper-proliferative and focally dysplastic. These lesions may evolve into tubular, tubulovillous or villous adenomas, with villous architecture associated with more rapid growth and a higher risk of malignant transformation, ultimately progressing to invasive carcinoma in a subset of cases [13].

For clarity, we use the term ‘mutations’ to refer specifically to nucleotide-level variants, including single-nucleotide variants and small insertions or deletions, unless otherwise stated. Larger-scale genomic alterations are referred to as copy number alterations (CNAs), structural variants or chromosomal instability (CIN), depending on context. This distinction is important, as point mutations can initiate tumorigenesis, whereas large-scale chromosomal alterations, particularly in combination with driver mutations, are critical for invasive potential and metastatic competence.

The initial multistep model of carcinogenesis describes the linear accumulation of somatic mutations and chromosomal instability, known as either the classic adenoma–carcinoma pathway or chromosomal instability (CIN) pathway [33]. This pathway, responsible for approximately 70–85% of colorectal cancers, typically begins with a loss-of-function mutation in the *APC* gene, with subsequent mutations in *KRAS*, *TP53*, and *SMAD4* [34].

An alternative mechanism of CRC development is the microsatellite instability (MSI) pathway which accounts for 12–15% of sporadic cancers [35]. The MSI pathway is caused by accumulation of mutations in microsatellite regions, most frequently through promotor methylation of the *MLH1* gene. The MSI pathway and the CIN pathway are often considered distinct evolutionary pathways, though not strictly mutually exclusive.

**Figure 2:**
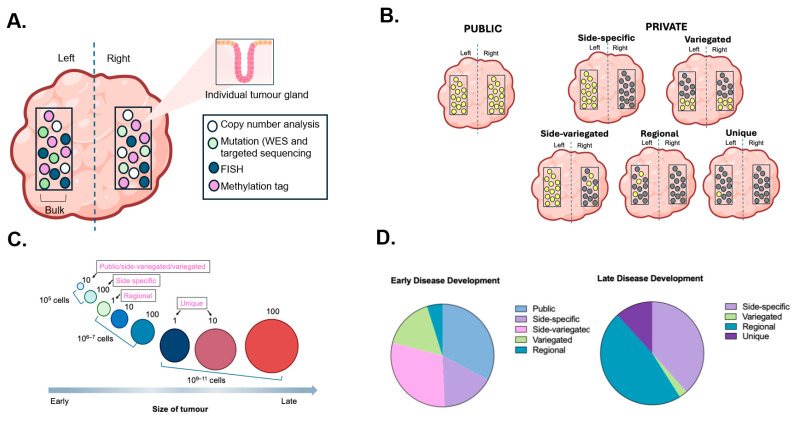
Big Bang model of human colorectal cancer (adapted from [28]). (**A**) Individual tumour glands and bulk tumour can be sampled from regions across the entire tumour. Using several different genomic techniques including copy number analysis, whole-exome sequencing (WES), and targeted sequencing, neutral methylation tag sequencing, and fluorescence in situ hybridisation test (FISH). (**B**) Spatial patterns can be grouped according to where the copy number alterations are detected in the tumour. Copy number alterations detected in all glands of the tumour are known as ‘public events’, and those found in specific regions of the tumour are known as ‘private events’ which are further subclassified as follows: side-specific (those found in all glands from one tumour side only), side-variegated (those found in all glands on one side and some from the opposite side), variegated (those found in a subset of glands from both sides), regional (those found in more than one but not all glands from one side only), and unique (those found in a single gland). (**C**) A schematic representation of the tumour mutational timeline, which illustrates that early copy number alterations dominate in early tumour evolution. The majority of alterations occurring early in the genomic landscape are public or non-unique private events. The tumour size is used as a surrogate marker for time. (**D**) Illustration of relative contribute of public versus private events in early and late disease development.

Focusing on understanding the earliest events in the growth of a cancer is important to direct new therapeutic approaches in cancer prevention, detection, and treatment [10,36,37]. Given that ancestral history is recorded within tumour cell genomes, these early genetic changes are encoded in the cells of the fully developed cancer and can be detected through detailed, high-throughput sequencing. To this end, Sottoriva et al. sampled 349 tumour glands and bulk fragments from the left and right side of advanced adenomas and carcinomas, enabling highly sensitive detection of sub-clonal alterations [28] (Figure 2A). The investigators examined single-gland profiling to reveal spatial patterns in copy number alterations and demonstrated spatial patterns which could be grouped according to whether the copy number alterations (CNAs) were found in all glands of the tumour (public) or not (private) (Figure 2B). In this context, public alterations are those present in all sampled glands and are inferred to arise early, whereas private alterations are restricted to subsets of glands and reflect later evolutionary events. The private mutations were then sub-classified according to location found within the tumour: Side-specific/regional—found in glands from one tumour side only; Side-variegated—found in all glands from one side and some from the other side; Variegated—found in a subset of glands from both sides; Unique mutations—defined as those found in a single gland (this spatial classification underpins the inference of early sub-clonal mixing shown in Figure 2). Through CNA pattern comparison, it was noted that benign colorectal adenomas were characterised by side-specific and unique CNAs, meaning there was no spatial mixing observed. In contrast, most of the invasive carcinomas displayed the same private CNAs in individual glands from the opposite sides of the cancer (variegated or side-variegated), suggesting a dispersion of early private mutations to distant sites within the primary CRC. Whole-exome sequencing was undertaken, which also detected sub-clone mixing in the CRCs which was not seen in adenomas. Tumour phylogenies were compiled alongside computational modelling. This revealed patterns consistent with a ‘Big Bang’ expansion where cancers grow as a single expansion, populated by a large number of early-arising clones, rather than by the stepwise accumulation of driver mutations (Figure 2C,D).

Classical models of CRC evolution invoke stepwise acquisition of driver mutations with successive clonal sweeps. In contrast, the ‘Big Bang’ model describes an alternative growth dynamic in which multiple genetically distinct clones arise early after malignant transformation and expand with limited subsequent selection, resulting in early establishment of intratumour heterogeneity. Support for this interpretation comes from spatially resolved single-gland sequencing and phylogenetic modelling, in which variegated copy number alteration patterns, early sub-clonal mixing, and mutation frequency distributions are more consistent with early neutral expansion than with serial selective sweeps [28,38]. Importantly, these represent best-fit computational interpretations rather than direct observations of tumour growth. Big Bang-like dynamics appear prevalent in many sporadic CRCs, particularly microsatellite-stable tumours, but are not universal and may vary by molecular subtype, including accelerated dynamics reported in Lynch syndrome-associated cancers [24,26]. This ‘Big Bang’ expansion has also been described in other cancers, notably breast cancers and hepatocellular carcinoma [38,39,40].

From a clinical perspective, it is plausible that sub-clonal variegation and mixing may reflect more aggressive tumour behaviour, given that this pattern of heterogeneity was not detected in benign colorectal adenomas. It is also important to consider the timing of development of sub-clones—those that develop early are generally passengers and are tolerated, whereas those that develop late may be more likely to reflect treatment-resistant mutations that should not be underestimated.

#### Challenges in Inferring Clonal Evolution

While spatial sequencing and phylogenetic modelling have provided major insights into CRC evolution, it is important to appreciate that clonal architectures are model-based interpretations rather than direct observations. Sequencing depth influences sensitivity for low-frequency variants and, therefore, the ability to distinguish neutral drift from positive selection, with shallow coverage favouring early clonal events and deeper coverage increasing susceptibility to technical noise [8,9]. Variable tumour purity can be an issue and can be observed in the processing of samples for spatial transcriptomic analysis [41]. This can distort variant allele frequencies and copy number estimates which makes the data less reliable. Consequently, evolutionary models such as the ‘Big Bang’ reflect dominant patterns inferred from statistical tests rather than direct single-cell observations [28] and should be interpreted as such.

### 2.2. CRC Molecular Classifications

Given the differences observed in CRC tumour phenotype, research groups have focused on scrutinising molecular features to formulate classifications based on distinctive genomic features and gene expression profiles [42]. With time, these classifications have become increasingly sophisticated to help direct treatment stratification for patients with this disease.

The consensus molecular subtype (CMS) classification was one of the first to be established through bulk RNA sequencing. Four subtypes resulted from this analysis. The CSM1 subtype (approximately 14% of CRC), most commonly arising in the right colon, is defined by microsatellite instability (MSI) and high immunogenicity. The most common mutations in CMS1 are found in *MSH6*, *PTEN*, *ATM*, *RNF43*, and *TGFβR2* genes. The CMS2 canonical subtype is characterised by marked chromosomal instability (CIN) and commonly found in the distal colon. Activation of the WNT/β-catenin and MYC signalling is usually evident in CMS2 which is associated with a classical epithelial transcriptional profile. CMS3 accounts for 13% of CRC. The most prevalent mutations within this subtype comprise those found in *APC*, *KRAS*, *TP53*, and *PIK3CA* genes. CMS3 tumours are associated with pathways such as lipogenesis, glutamine metabolism, and metabolic dysregulation. CMS4 represents 23% of CRC and is associated with the worst prognosis. This subtype is characterised by epithelial–mesenchymal transition (EMT), stomal invasion, and angiogenesis. CMS4 is driven by transforming growth factor β (TGF-β) signalling [43].

Two years later, the Colorectal Intrinsic Subtypes (CRIS) were developed from CRC xenografts with the aim of defining cancer-cell-specific transcriptional signatures and exclusion of the stroma [44]. From this analysis, five subtypes were identified, CRIS-A to CRIS-E. CRIS-A was associated with mucinous tumours that were MSI or harboured *KRAS* mutations. CRIS-B subtypes were associated with a poor prognosis and driven by TGF-β pathway activity with epithelial-to-mesenchymal transition (EMT). CRIS-C had high EGFR signalling, and CRIS-D tumours were associated with WNT activation. Finally, CRIS-E harboured *TP53* mutations and had a Paneth cell-like phenotype [44].

Five years later, an intrinsic [iCMS] classifier was developed through single-cell sequencing of approximately 50,000 epithelial cells [45]. This analysis identified two epithelial classes with distinct genomic profiles. The iCMS2 class was associated with somatic copy number alterations [SCNA]/copy number variation (CNV) spread across difference chromosomal regions, whereas iCMS3 displayed limited uniformity in CNVs. However, all MSI tumours were classified as iCMS3. The investigators demonstrated that the iCMS system could be combined with the bulk CMS classification such that CMS4 could be further grouped according to prognosis; iCMS2 (better outcome) and iCMS3 (worse outcome). Overall, iCMS3 tumours were more likely to be associated with *BRAF*, *KRAS*, and *PIK3CA* mutations, whereas iCMS2 tumours were associated with mutations in *APC* and *TP53* [45].

Most recently in 2024, an alternative approach was undertaken whereby three pathway-derived subtypes (PDS) in CRC were identified. PDS1 comprised fast cycling canonical stem cells; PDS2 was associated with regenerative stem cells. PDS3 was associated with low proliferative markers and lacked stem cells. Interestingly, when CMS and PDS classifications were compared within the same samples, it became clear that the CMS2 group could be split into PDS1 and PDS3. Furthermore, CMS3/CMS4 subtypes could be combined within PDS2 [46].

## 3. Phenotypic Plasticity in CRC

### 3.1. Phenotypic Plasticity in Localised CRC

The functional attributes of cancer cells should be carefully considered as they have enormous impact on cancer care and outcomes. As cancers progress, they acquire resistance to successive lines of therapy. However, somatic mutations are largely conserved between primary and metastatic tumours from the same patients, suggesting that non-genetic phenotypic plasticity has an important role in cancer progression and treatment resistance [20,47,48]. Phenotypic plasticity also arises from the regulation of transcription factor networks downstream of microenvironment-responsive-signalling pathways. Modulation of WNT, TGF-β and NOTCH signalling enables reversible transitions between differentiated, stem-like and progenitor states [49,50,51]. These transitions are further supported by epigenetic plasticity, which maintains permissive chromatin states that allow transcriptional reprogramming in response to environmental cues [18].

To apprehend the relationship between CRC cell phenotype and the accumulation of genetic mutations, Househam et al. undertook paired DNA and RNA sequencing of single CRC glands from multiple areas within CRC tumours [18]. First, they explored the heterogeneity of gene expression by grouping the data according to level of expression and degree of variance within the samples. Their analyses confirmed that gene expression pathways known to be important for cancer cell survival and interaction with the microenvironment were not uniformly expressed across CRC tumours. They also set out to define the genetic determinants of gene expression variance, as understanding whether gene expression is genetically encoded or environmentally driven has implications for treatment targeting and resistance prediction. They reasoned that if transcriptional heterogeneity is caused by genetic heterogeneity, then gene expression variability should mirror genetic ancestry. In fact, this comparison demonstrated that the expression of most genes did not display a pattern of heritability. Instead, transcriptional heterogeneity is most likely to be influenced by external stimuli from the tumour microenvironment, a concept defined as ‘phenotypic plasticity’. Although the expression of a small proportion of genes was found to be associated with CNAs and mutations, most somatic mutations did not result in a large change in gene expression. As expected, the most frequently mutated known drivers of CRC including *APC*, *KRAS*, *TP53*, *PTEN*, *EGFR*, and *ATM* were largely clonal. However, the majority of other candidate sub-clonal driver mutations showed no evidence of a survival advantage, which is in keeping with published reports of widespread neutral sub-clonal evolution within CRCs [18]. These data confirm that transcriptional heterogeneity is common within and between CRCs. Less than one percent of expressed genes with sub-clonal gene expression variation showed compelling evidence of genetic heritability. These data strongly support phenotypic plasticity as a common occurrence that is responsible for transcriptional heterogeneity in CRC [18].

### 3.2. Increased Phenotypic Plasticity in Metastatic CRC

To study metastatic progression in CRC patients, Moorman et al. analysed matched trios of normal colon, primary colorectal cancer, and metastatic tissues from patients undergoing synchronous hemicolectomy and metastasectomy, including both treatment-naïve patients and those who received pre-operative chemotherapy [51].

The investigators found that CRC progression involved three distinct, ordered cell state transitions: (1) from differentiated intestinal states in normal colon to a LGR5 intestinal stem cell (ISC)-like state enriched in the primary tumour; (2) developmental reprogramming to a highly plastic progenitor-like foetal state associated with epithelial injury; and (3) expression of non-intestinal lineage gene programmes, including squamous and neuroendocrine, which are enriched in metastases. Organoids derived from profiled trios revealed that metastatic cells possess greater cell-intrinsic plasticity in vitro relative to primary tumour cells from the same patient, allowing them to adapt to distinct microenvironments in the colon and liver in vivo. In this study, primary CRC cells were more likely to remain in intestinal lineage than matched metastatic cells. In part, this was due to the repressor activity of *PROX1*. Loss of *PROX1*-dependent lineage restriction during tumour progression resulted in differentiation into non-canonical lineages. This data supports a two-stage model of metastatic plasticity, whereby metastasis promotes highly plastic cell states that can be induced to differentiate along diverse trajectories by cues from the tumour microenvironment [51].

While these studies provide important insights into phenotypic plasticity in CRC, limitations include relatively small patient cohorts, incomplete sampling of tumour regions, and technical constraints of single-cell RNA sequencing including transcript capture and gene dropout. Current spatial transcriptomic approaches lack single-cell resolution—but the consistency of data across independent studies supports the influence of phenotypic plasticity in CRC.

## 4. Interactions of the Genome and Epigenome in CRC

### 4.1. Epigenetic Modifications in CRC Progression

**Figure 3:**
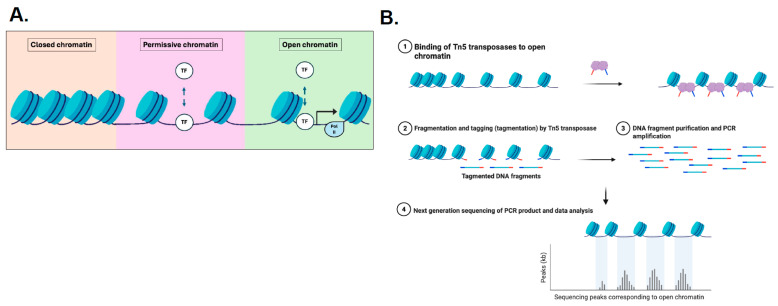
Co-evolution of the genome and epigenome in CRC (adapted from [54]). (**A**) The continuum of chromatin states across the genome is dynamic and reflects the regulatory capacity of the epigenome. In comparison to closed chromatin, permissive chromatin is dynamic and allows for transcription factors (TF) to initiate remodelling of chromatin and establish an open chromatin formation (as seen here for an active gene locus). Pol II, RNA Polymerase II; TF, Transcription factor. (**B**) Quantification of chromatin accessibility using ATAC-seq.

In this review, we have discussed the valuable role of comprehensive, spatially resolved DNA and RNA sequencing in deciphering CRC cell biology and clonal evolution. We know, however, that epigenetic changes can also be responsible for phenotypic variation between cancer cells. Chromatin, a complex of histones that make up chromosomes, packages long DNA molecules in a compact form that allows it to fit inside the nucleus [52,53]. Chromatin plays an important role in gene expression by regulating which genes are accessible for gene expression. Therefore, chromatin accessibility, the ability of nuclear macromolecules to physically contact chromatinised DNA, also needs to be evaluated since the landscape of chromatin accessibility reflects transcriptional regulation [54,55] (Figure 3A).

In CRC, permissive chromatin states are thought to underpin phenotypic plasticity by maintaining regulatory regions in a transcriptionally primed configuration, allowing cancer cells to rapidly adopt stem-like, mesenchymal, or immune-evasive phenotypes in response to microenvironmental signals. Spatial multi-omic studies show that such permissive states can exist independently of stable genetic alterations, supporting a model in which epigenetic priming enables dynamic cell state transitions without requiring new mutations [17].

DNA methylation at CpG islands within promotor regions, associated with transcriptional repression, has been studied in CRC [56,57]. The CpG island promotor methylation phenotype (CIMP) is a distinct molecular subtype of CRC characterised by widespread hypermethylation of CpG islands and is strongly associated with *BRAF* mutations and a poorer outcome. In contrast, DNA promotor methylation can occur in specific tumour suppressor genes, leading to gene silencing and disruption of cellular growth regulation and cell cycle checkpoints [58]. This is evidenced by MLH1 in which aberrant promotor hypermethylation leads to MSI which is associated with an improved prognosis and an increased chance of response to immune check point inhibition [59]. Genome-wide hypomethylation, on the other hand, can drive tumour progression through its contribution to chromosomal instability, increased mutation rate, and activation of oncogenic pathways such as Wnt/β-catenin, TGF-β, and PI3K/AKT/mTOR pathways [60,61].

### 4.2. Co-Evolution of the Genome and Epigenome

Advances in technology have allowed the measurement of chromatin accessibility using small quantities of DNA. It can be measured via high throughput by quantifying the susceptibility of chromatin to cleavage of its constituent DNA. For example, an assay for transposase-accessible chromatin using sequencing [ATAC-seq] uses a hyperactive Tn5 transposase to simultaneously cleave and ligate adaptors to accessible DNA. ATAC-seq then selectively amplifies double cleavage events, and libraries can be generated in less than 2 h [54,62,63] (Figure 3B).

With respect to CRC, Heide et al. undertook spatial multi-omic sequencing of individual crypts, including whole-genome sequencing and RNA-seq as well as ATAC-seq [17]. Given that DNA mutations alone do not fully explain malignant transformation, the authors explored the relationship (co-evolution) of the genome and epigenome. In this study, somatic mutations of chromatin modifier genes were analysed to investigate the influence of genetic mutations on the epigenome. Clonal truncating mutations in chromatin modifier genes of microsatellite stable (MSS) cases showed clear signs of positive selection. Although sub-clonal chromatin modifier mutations were also present, positive selection was not detected here. There was also no positive selection detected in microsatellite unstable cancers. The landscape of somatic chromatin accessibility alterations (SCAAs), as described above, were evaluated, revealing highly recurrent, largely clonal SCAAs in both promotors and enhancers for numerous cancer-related genes. Many of these genes did not harbour genetic mutations, suggesting that SCAAs are an alternative mechanism for driver gene regulation. In addition, most recurrent SCAAs were not found in adenomas, implying that most SCAAs occurred at the onset of malignant transformation but before sub-clonal diversification. The impact of SCAAs on gene expression was assessed using matched RNA sequencing; approximately 11% of promotors and 13.5% of enhancers with recurrent SCAAs showed signs of altering the expression of associated genes. In addition, evidence of reactivation of developmental genes was observed. Although this needs to be further functionally validated, it is possible that epigenetic alterations may be responsible for reprogramming cell fate in CRC cells. Overall, non-genetic determinants of cancer cell biology and clonal evolution are important in CRC regulation [17].

## 5. Genome and Epigenome Regulation of the Immune Environment in CRC

### 5.1. The Immune Environment in CRC Progression

**Figure 4:**
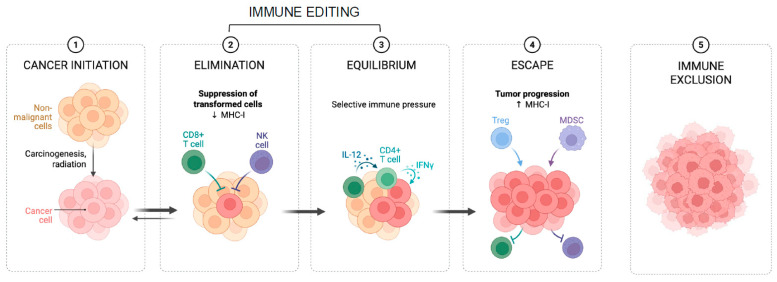
Cancer immunoediting (adapted from [71]). Tumours (panel 1) circumvent immune destruction by immune editing (losing antigenic mutations (panels 2 and 3)), immune escape (develops mechanisms to avoid detection) (panel 4), and immune exclusion, where immune cells are unable to infiltrate the cancer, leading to ineffective anti-tumour responses (panel 5).

The immune system plays a pivotal role in detecting and killing cancer cells [64,65,66]. Neoantigens are newly formed peptides generated by cancer-specific antigenic mutations that allow the recognition of cancer cells by the immune system [67,68,69,70]. Tumours can circumvent immune destruction by immune editing (losing antigenic mutations), immune escape (developing mechanisms to avoid detection), and immune exclusion, where immune cells are unable to infiltrate the cancer leading to ineffective anti-tumour responses [71,72,73] (Figure 4). Immune escape is important for mismatch repair-deficient (MMRd) CRC development [64,74,75].

CRC progression and outcome are strongly influenced by changes in the immune microenvironment. Immune cell infiltration has emerged as a reliable prognostic indicator, and this has led to the development of the Immunoscore, which quantifies T cell infiltration in primary CRC and serves as an independent predictor of distant relapse [76]. By combining digital pathology and immunohistochemical analysis, the Immunoscore measures the density of CD3^+^ and CD8^+^ T lymphocytes in both the tumour core and the invasive margin. This scoring system ranges from Immunoscore 0 (low immune density) to Immunoscore 4 (high immune density), offering insight into tumour immunogenicity. Tumours with higher Immunoscores are typically more immunogenic, often correlating with a greater neoantigen load and improved prognosis [77]. However, tumours with lower immunoscores are more likely to metastasise [78].

A proportion of MMRd CRCs are associated with a higher neoantigen burden and enhanced immune presence. However, approximately 30% of MMRd CRCs do not respond to immune checkpoint blockade therapies, and mismatch repair-proficient (MMRp) CRCs display a lower mutational burden [74,79,80]. Furthermore, we understand from other tumour types such as melanoma and non-small cell lung cancer that most tumour antigens are epigenetically regulated and encoded by noncanonical genomic sequences (so-called ‘dark antigens’), with computational approaches increasingly important for neoantigen prediction and clinical translation [80].

While evolutionary and epigenetic mechanisms clearly influence therapeutic resistance, their clinical translation remains limited. At present, most approaches for identifying epigenetically regulated tumour states or so-called ‘dark antigens’ rely on high-depth sequencing, specialised computational pipelines, and extensive annotation [74]. This restricts their use largely to research settings. Although bioinformatic frameworks for neoantigen prediction and epigenomic profiling are rapidly advancing, including methods integrating chromatin accessibility and non-canonical transcript sources, these are not yet routinely deployable within standard clinical timelines. Bridging this translational gap will require streamlined assays, validated biomarkers, and prospective studies demonstrating clinical utility beyond genomic profiling alone.

### 5.2. Genome and Epigenome Regulation of CRC Immune Environment

Until recently, the evolutionary dynamics of immune control in CRC have been largely uncharacterised. However, single-cell sequencing has enabled the construction of a comprehensive CRC immune atlas in which 33 immune cell clusters were identified, with cell composition patterns classified into five distinct tumour microenvironment subtypes [81,82]. There has also been a paucity of knowledge relating to how immune dynamics shapes the epigenome. To address this, Lakatos et al. analysed spatially resolved neoantigen data from individual tumour glands through imaging the tumour microenvironment (TME) as well as SCAAs. They noted that the proportion of PD-L1+ tumour cells, known to inhibit T cell-mediated killing by binding PD-1 receptors, was significantly higher at the invasive margin, supporting heightened immune surveillance at this tumour site. In this study, clonal high-impact immune escape alterations were associated with loss of function in antigen presentation. Through phylogenetic signal analysis, the authors confirmed that the expression of antigen-presenting genes (APGs) is plastic rather than heritable. Frequent loss of accessibility at APGs was observed, and SCAAs were confirmed to contribute to neoantigen silencing, leading to decreased immune recognition and facilitating immune escape, even in the absence of genetic mutations. Furthermore, somatic mutations with the highest predicted impact on immune evasion were mostly shared across the whole tumour, indicating they occur early at the onset of CRC formation, consistent with a ‘Big Bang’ evolutionary pattern [19,28].

Mechanistically, signalling pathways such as Wnt/β-catenin, TGF-β, and PI3K/AKT have been implicated in shaping the tumour immune microenvironment by promoting immune suppression, T cell exclusion, and epigenetic remodelling [83]. Taken together, it is apparent that epigenetic, transcriptomic, and microenvironmental mechanisms regulate the CRC immunogenomic machinery. From a therapeutic perspective, this data supports the exploration of disrupting epigenetic pathways as a novel strategy to enhance the response to immunotherapy in CRC [34].

## 6. Discussion

In this review, we discuss studies that illustrate how the complex interplay between the genome, epigenome, and the immune system in CRC can be unravelled through advancements in high-resolution, spatially resolved sequencing and mathematical modelling [17,18,19,27,28]. Instead of the linear accumulation of genetic drivers as the predominant cause of cancer progression, we can see how CRCs grow as a single expansion populated by early-arising clones [19,28].

We now know that transcriptional heterogeneity is common within and between CRCs, and the quantified change in gene function regulation by the microenvironment allows us to appreciate the importance of phenotypic plasticity in CRC behaviour [18]. There is also compelling evidence that non-genetic determinants, such as epigenetic regulation, are influential in shaping CRC cell biology and clonal evolution [54]. Furthermore, it is highly plausible that the combination of epigenetic, transcriptomic, and microenvironmental mechanisms regulate CRC immunogenomic machinery to facilitate CRC immune evasion [71].

Altogether, these studies underscore that the evolutionary trajectory of colorectal cancer is not simply a product of mutational burden but a reflection of dynamic interactions between genetics, epigenetics, and the tumour microenvironment. Further functional validation studies are crucial, particularly those exploring how plasticity and epigenetic reprogramming may be reversed or targeted therapeutically.

The data presented gives us a glimpse into the elaborate evolutionary systems that can influence not only tumour progression but also resistance to anticancer therapies. Such evolutionary dynamics increase the likelihood that rare genetic sub-clones or adaptive cell states exist before treatment, providing a basis for anticancer therapies. It is only through studies such as those presented in this review that we can pinpoint cellular pathways that could be exploited to enhance treatment response and circumvent resistance.

## Data Availability

No new data were created or analysed in this study. Data sharing is not applicable.
